# Update to the study protocol *Face Your Fears: Virtual reality-based cognitive behavioral therapy (VR-CBT) versus standard CBT for paranoid ideations in patients with schizophrenia spectrum disorders: a randomized clinical trial*

**DOI:** 10.1186/s13063-023-07069-7

**Published:** 2023-01-23

**Authors:** U. N. Jeppesen, A. S. Due, L. Mariegaard, A. Pinkham, M. Vos, W. Veling, M. Nordentoft, L. B. Glenthøj

**Affiliations:** 1 Copenhagen Research Centre On Mental Health (CORE), University of Copenhagen, Copenhagen, Denmark; 2 Department of Psychology (DK), University of Copenhagen, Copenhagen, Denmark; 3 School of Behavioral and Brain Sciences, University of Texas at Dallas, Richardson, USA; 4 Faculty of Medical Sciences, University Medical Center Groningen, Center of Psychiatry, University of Groningen, Groningen, The Netherlands

**Keywords:** Schizophrenia spectrum disorders, Schizotypal disorders, Delusions, Paranoid ideations, Ideas of reference, Cognitive behavioral therapy, Virtual reality exposure therapy, Virtual reality, Social functioning, Activities of daily living

## Abstract

We unfortunately need to make an update to our published study protocol that describes a significant change in the design of the study. The Committee on Health Research Ethics of the Capital Region Denmark recently rejected the approval of changing the primary outcome in the trial, on the invariable grounds that the trial has already commenced. It is therefore necessary to retain the Green Paranoid Thought Scale (GPTS) part B, ideas of persecution, as our primary outcome, and GPTS part A, ideas of social reference, as a secondary outcome, which is described opposite in our published study protocol. The exchange of outcomes has not affected participation in our trial or the informed consent. Intervention in both groups and assessments are unchanged. The two outcomes together constitute GPTS and the unifying concept we attempt to treat, namely paranoid ideations. As this is a blinded, methodologically rigorous trial, we did not have—and still do not have—access to preliminary data, and therefore, we have no knowledge of the distribution of our two intervention groups nor the potential effect of the intervention. The power calculation remains unchanged irrespective of the selection of the primary outcome. We have been fully transparent with the changes in primary and secondary outcomes on ClinicalTrials.gov throughout the trial. Due to the considerations mentioned above, we assumed that there would not be any ethical implications of the change of primary outcome. We sincerely apologize for the irregularity caused because of this assumption.

**Trial registration**

ClinicalTrials.gov NCT04902066. Initial release April 19th, 2021.

## Main text

We unfortunately need to make an update to our published study protocol [1] identifying a significant change in the design, as we are forced to change back to our originally planned primary outcome.

In a population of patients diagnosed with schizophrenia spectrum disorders (ICD-10, F20-29, schizotypal disorder included), who receive treatment as usual, we are investigating if the effect of virtual reality-based cognitive behavioral therapy (VR-CBT) is superior compared to cognitive behavioral therapy (CBT), as add-on treatment focusing specifically on paranoid ideations (encompassing ideas of social reference and manifest persecutory delusions); in reducing paranoid ideations, social anxiety, avoidance and safety behavior; and in improving social cognition, psychosocial functioning, and quality of life. This is investigated in a randomized, assessor-blinded parallel-groups multi-center superiority clinical trial, fulfilling the CONSORT criteria for non-pharmacological treatment where participants are assessed at baseline, treatment end (3 months post-baseline), and then 9 months post-baseline.

After being forced to change back to our originally planned primary outcome, our primary hypothesis is:

- VR-CBT will be superior to CBT in reducing ideas of persecution, in patients with schizophrenia spectrum disorders (ICD-10, F20-29, schizotypal disorder included).

When our trial was initiated, the original primary outcome was ideas of persecution, measured with part B in the Green Paranoid Thought Scale (GPTS) while ideas of social reference, measured with part A in GPTS, were listed as a secondary outcome in the trial protocol, on ClinicalTrials.gov and in the approval from of the Committee on Health Research Ethics of the Capital Region Denmark.

During the trial, our impressions of the clinical assessments were that ideas of social reference seem to be a more appropriate primary outcome due to our population including people diagnosed with schizotypal disorders along with participants with manifest psychotic disorders.

We observed that participants with schizotypal disorders experiencing ideas of social reference, which are more attenuated paranoid ideations, would often receive a low score on the GPTS part B. Therefore, listing GPTS part B as the primary outcome would hypothetically only reflect the symptom level of part of the study population (patients with manifest psychosis), while not fully comprising the symptom level, or fully capturing potential for change, found in the population of patients with schizotypal disorder.

As of February 23, 2022, 10 months into the trial, wherein 79 out of 256 participants were included and had participated in baseline assessments, we decided after thorough consideration to exchange our primary outcome, GPTS part B, ideas of persecution, with our secondary outcome, GPTS part A, ideas of social reference, as this was intended to capture the symptom level in the total study population.

The exchange did not affect participation in our trial or the informed consent. Intervention in both groups and assessments were unchanged. The two outcomes together constitute the GPTS and the unifying concept we attempt to treat, namely paranoid ideations. As this is a blinded, methodologically rigorous trial, we had not had (and still do not have throughout the study period) access to preliminary data. Therefore, we have no knowledge of the distribution of our two intervention groups nor the potential effect of the intervention.

The power calculation remains unchanged irrespective of the selection of the primary outcome. (Ideas of persecution: clinically relevant difference 6.0, SD 17.9, *N* = 128*2, power = 80% versus ideas of social reference: clinically relevant difference 5.5, SD 15.5, *N* = 128′2, power = 81%). Due to the factors mentioned above, we did not find any reasons for ethical implications of the change of primary outcome—as we also were fully transparent with this change of outcome on ClinicalTrials.gov.

We therefore assumed that the ethical committee would approve this change. However, on September 3, 2022, we received a rejection from the Committee on Health Research Ethics of the Capital Region Denmark on changing outcomes, on the invariable grounds that the trial had already commenced. This means that it is necessary to retain GPTS, part B, ideas of persecution, as our primary outcome and GPTS, part A, ideas of social reference, as a secondary outcome.

Changing the primary outcome back and forth has not affected our ongoing clinical trial. However, we did publish our study protocol before the final decision of the ethical committee, and we sincerely apologize for the irregularity caused because of the above mentioned assumption.

## Data Availability

The datasets from the study will be available from the corresponding author upon reasonable request.

## Abbreviations

GPTS: Green Paranoid Thought Scale
VR-CBT: Virtual Reality-based Cognitive Behavioral Therapy
CBT: Cognitive Behavioral Therapy
